# Supplementary material for: A tin fluoride-free, efficient and durable tin-lead perovskite solar cell
Source: Nat Commun. 2026 Jan 12;17:360. doi: 10.1038/s41467-025-65445-0 (PMC12796181; doi:10.1038/s41467-025-65445-0)
Supplement: Supplementary file 1 — Supplementary Information [file 41467_2025_65445_MOESM1_ESM.pdf]

Supplementary Information for

A Tin Fluoride-Free, Efficient and Durable Tin-Lead Perovskite Solar Cell

Haobo Yuan[1]†, Wenxiao Zhang[1]†*, Feng Wang[2], Jianhong Xu[1], Yuyang Hu[1], Xuemin Guo[1], Yunfei Li[1], Bo Feng[1], Zhengbo Cui[1], Wen Li[1], Sheng Fu[1], Xiaodong Li[1], Feng Gao[2]*, Junfeng Fang[1]*

[1]School of Physics and Electronic Science, Engineering Research Center of Nanophotonics & Advanced Instrument, Ministry of Education, East China Normal University, Shanghai 200241, China;

[2]Department of Physics, Chemistry and Biology (IFM), Linköping University, Linköping, Sweden.

*E-mail: jffang@phy.ecnu.edu.cn; feng.gao@liu.se; wxzhang@phy.ecnu.edu.cn

† These authors contributed equally to this work.

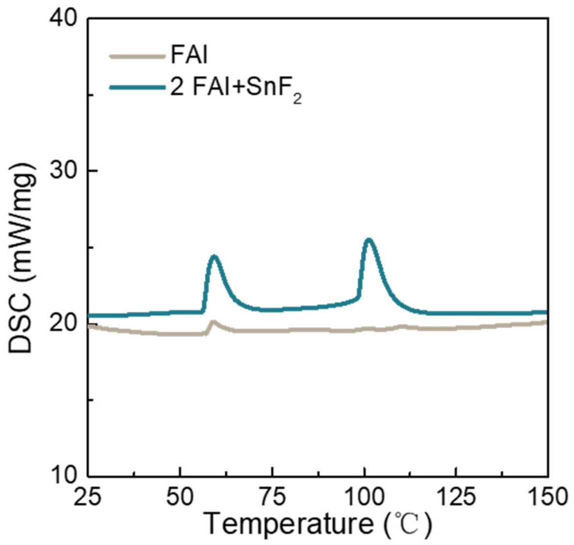

Supplementary Figure 1. DSC properties of FAI and FAI+SnF_2 powders.

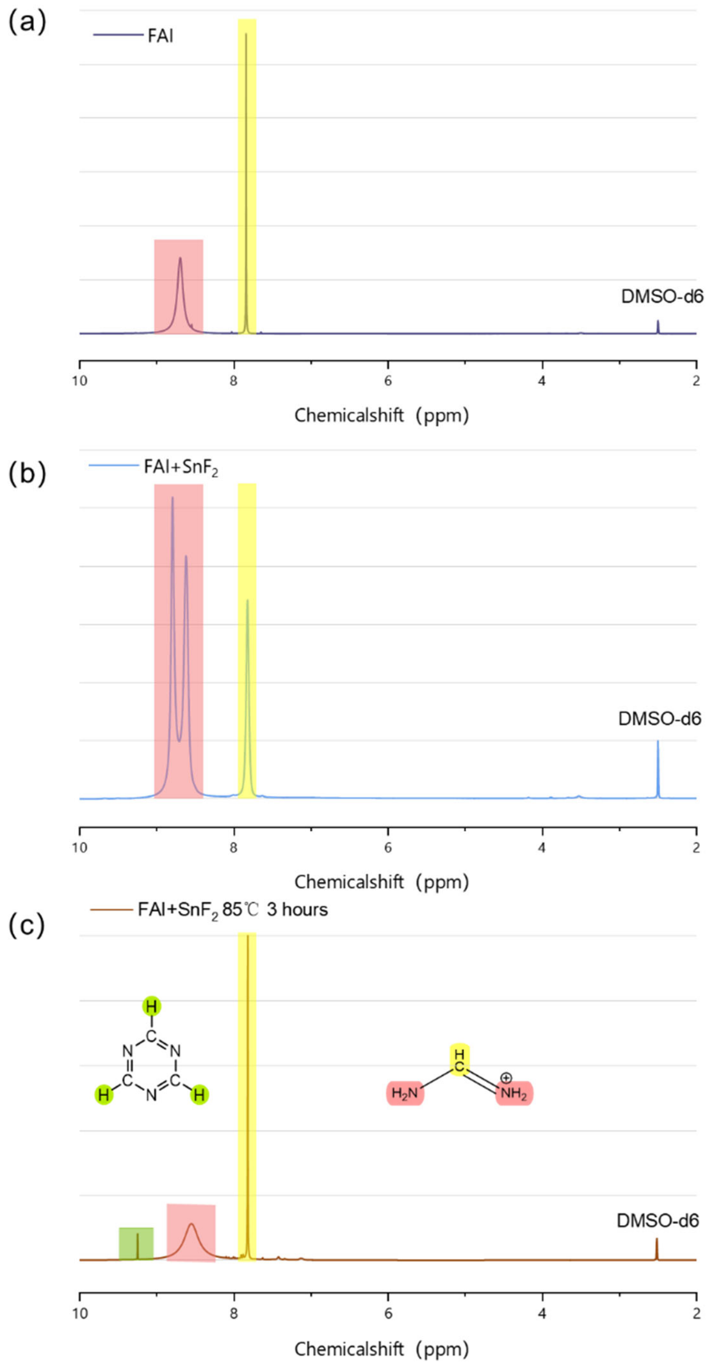

Supplementary Figure 2. The full ^{1}H NMR spectra of (a) FAI, (b) FAI+SnF_2 and (c) FAI+SnF_2 aging at 85°C for 3 hours.

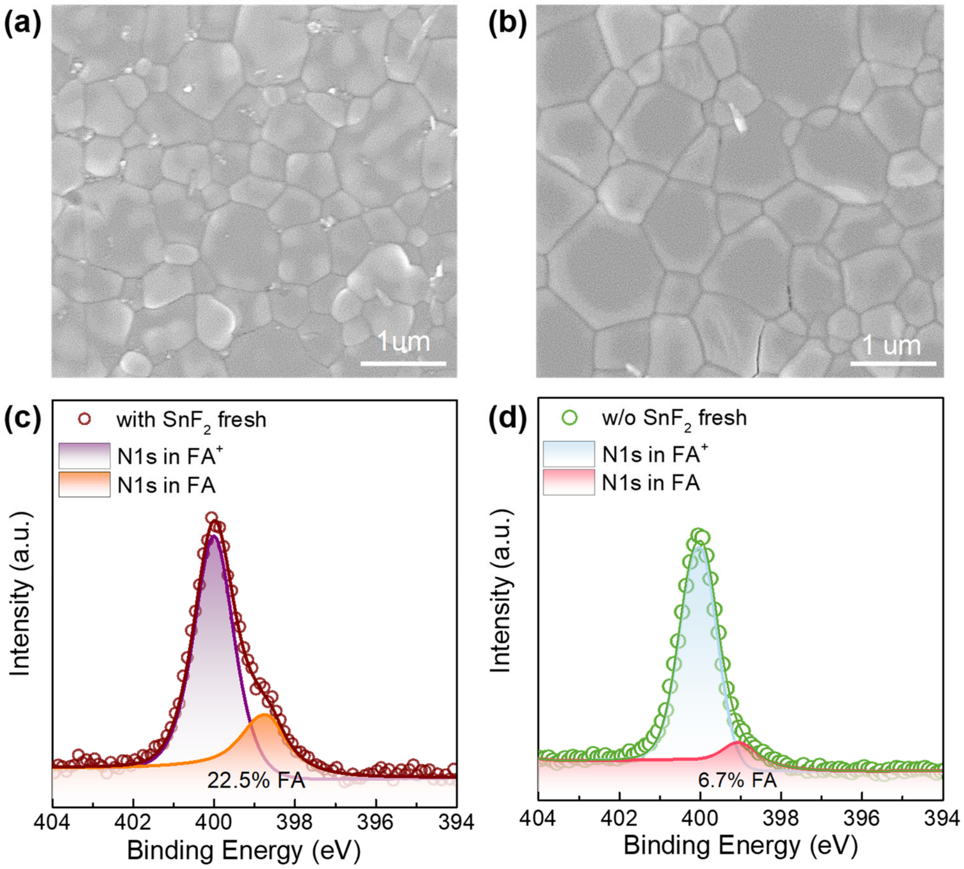

Supplementary Figure 3. The perovskite sample before the heat-photic aging. (a-b) SEM spectra of fresh Sn-Pb perovskite film with and without SnF_2 additive; (c-d) N *1s* XPS spectra of fresh Sn-Pb perovskite film with and without SnF_2 additive.

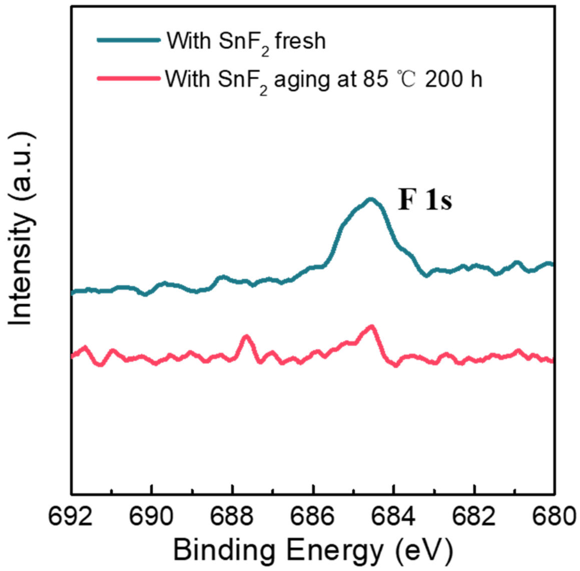

Supplementary Figure 4. F *1s* XPS spectra of Sn-Pb perovskite with SnF_2 additive before and after aging at 85 °C with light soaking for 200 h.

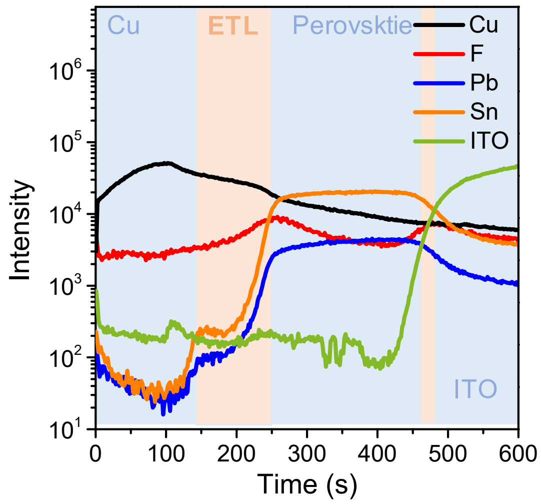

Supplementary Figure 5. TOF-SIMS depth profile of Sn-Pb perovskite film with SnF_2 additive after aging at 85°C under MPP condition for 200 h. The related ions to tin, lead, Cu and ITO are $[SnI_2]^-$, $[PbI_2]^-$, $[Cu]^-$ and $[InO]^-$ with m/z of 372.53, 461.86, 62.93 and 130.94, respectively.

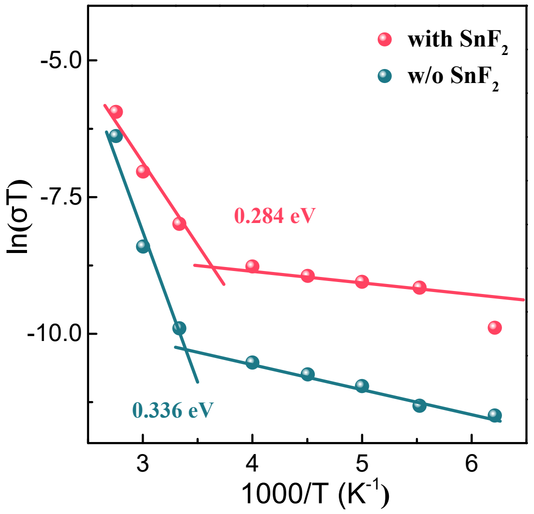

Supplementary Figure 6. The temperature-dependent conductivity of perovskite films with and without SnF_2 additive.

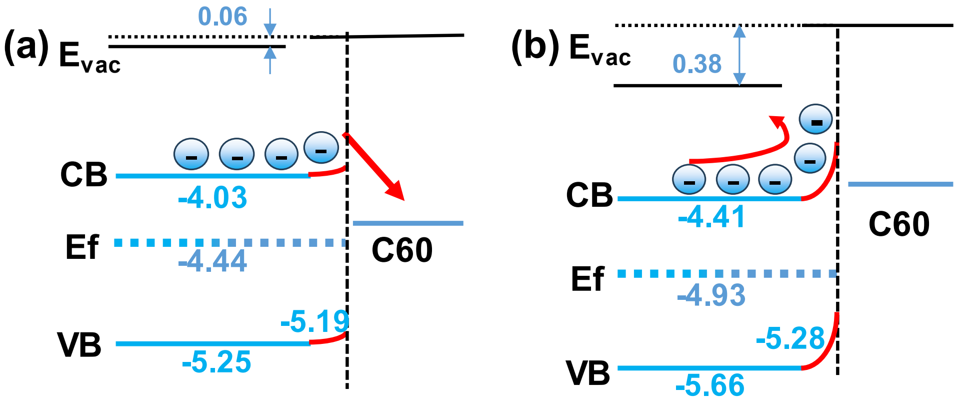

Supplementary Figure 7. The energy band regulation dilemma caused by SnF_2. (a) The energy band of perovskite/C60 interface with SnF_2 additive before aging. (b) The energy band of perovskite/C60 interface without SnF_2 additive befogre and after aging at 85℃ with light soaking for 200 h.

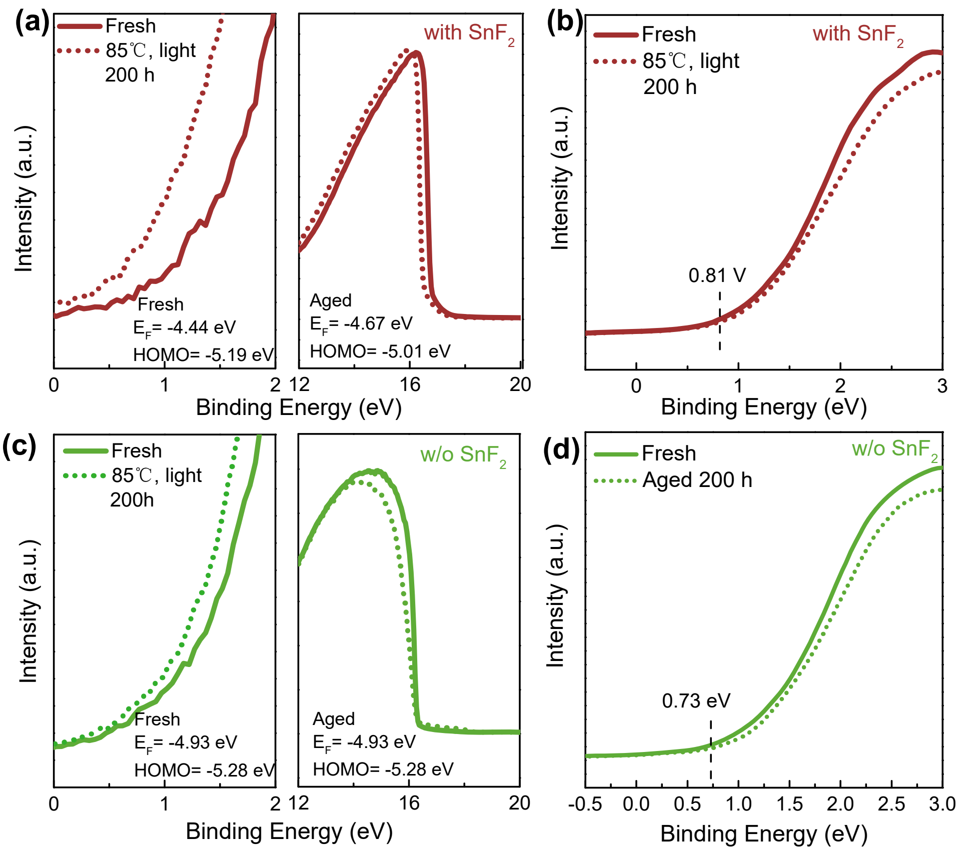

Supplementary Figure 8. The energy band changing caused by heat-photic aging. (a) Ultraviolet photoelectron spectroscopy (UPS) of Sn-Pb perovskite with SnF_2 additive before and after aging at 85°C with light soaking for 200 h. (b) Valence band spectra of Sn-Pb perovskite with SnF_2 additive before and after aging at 85°C with light soaking for 200 h. Valence band spectra is used to delineate the valence band-fermi level offset in the deeper region beneath perovskite film surface. (c) UPS spectra of Sn-Pb perovskite without SnF_2 additive before and after aging at 85°C with light soaking for 200 h. (d) Valence band spectra of Sn-Pb perovskite without SnF_2 additive before and after aging at 85°C with light soaking for 200 h.

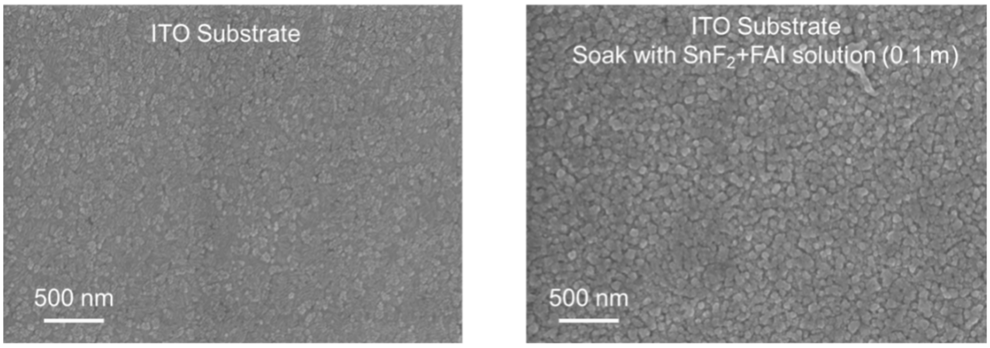

Supplementary Figure 9. SEM of ITO substrate before and after 48 h immersion in a 0.1 M SnF_2/FAI mixed solution followed by DMF rinsing.

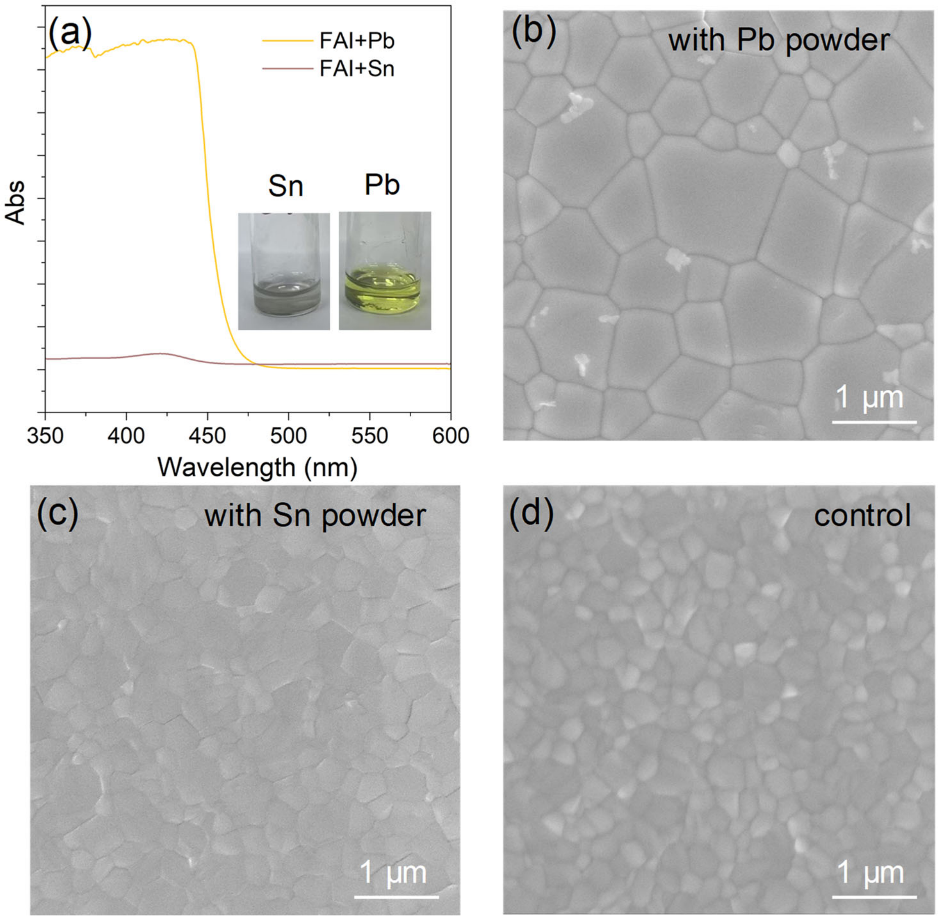

Supplementary Figure 10. The reducibility and crystalline modification function of the Pb powder. (a) The UV-vis absorption spectra of FAI solution with lead powder or tin powder. (b-d) The SEM of Sn-Pb perovskite film derived from precursors with (b) lead powder, (c) tin powder and (d) no metal powder (control).

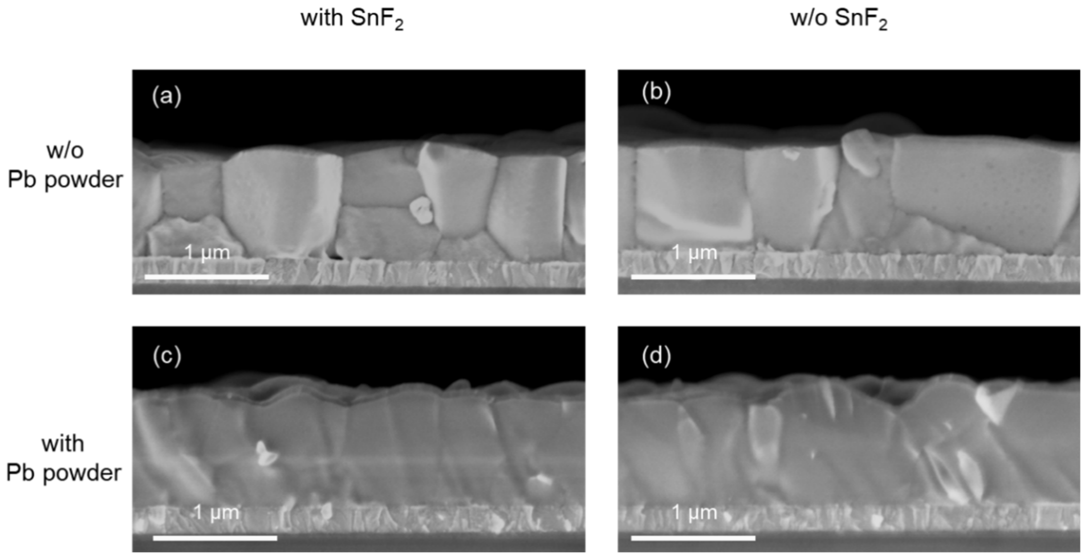

Supplementary Figure 11. The cross-SEM of Sn-Pb perovskite film in the absence of Pb powder or SnF_2 additive. The sample without Pb powder (a) with SnF_2 and (b) without SnF_2. The sample with Pb powder (c) with SnF_2 and (d) without SnF_2.

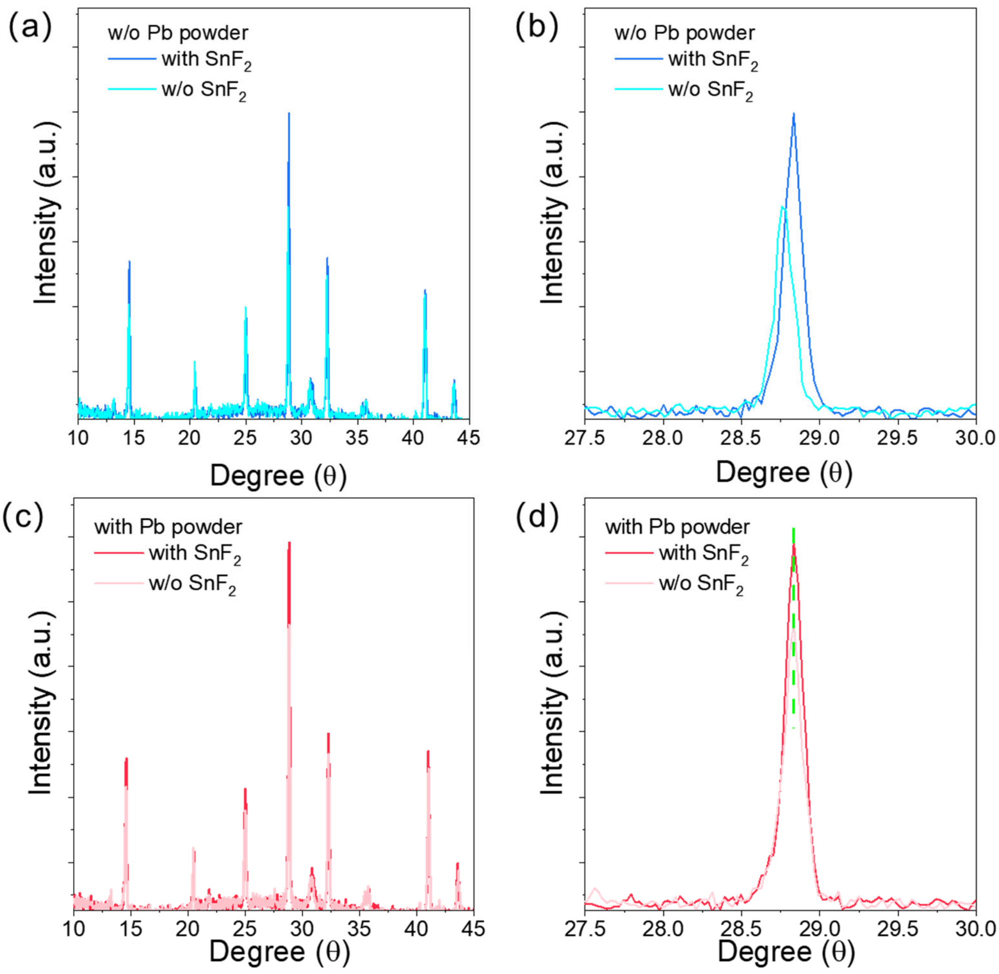

Supplementary Figure 12. The XRD patterns of Sn-Pb perovskite film in the absence of Pb powder or SnF_2 additive. The shift of XRD peak is caused by the V_{Sn} in perovskite lattice. The sample without Pb powder (a) with SnF_2 and (b) without SnF_2. The sample with Pb powder (c) with SnF_2 and (d) without SnF_2.

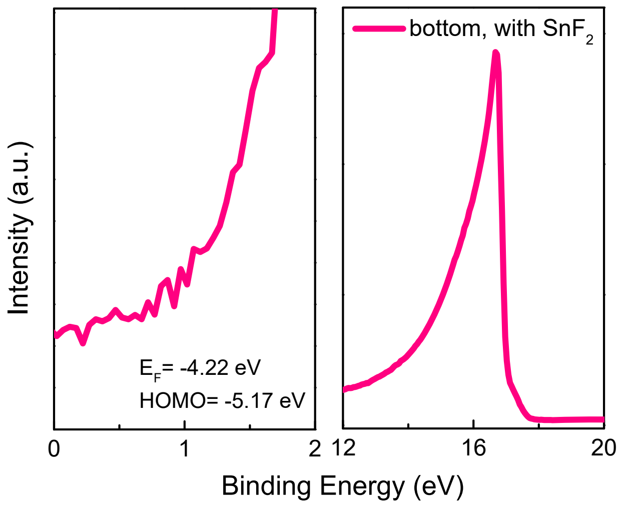

Supplementary Figure 13. The UPS spectra of the bottom surface of fresh Sn-Pb perovskite films with SnF_2 additive.

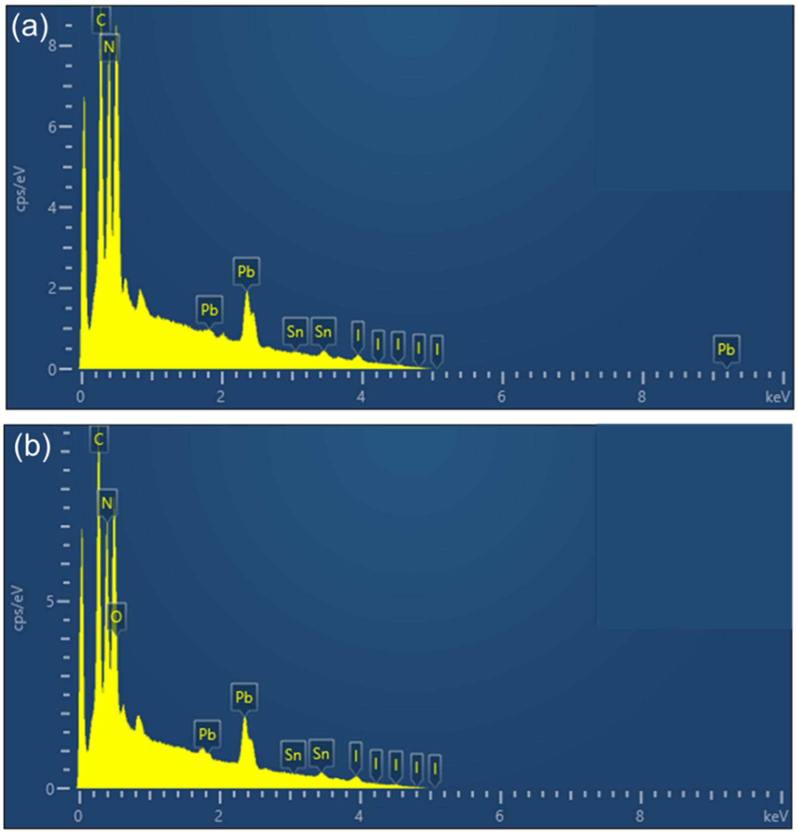

Supplementary Figure 14. The Energy Dispersive X-Ray Spectroscopy maps of perovskite films: (a) pristine and (b) after PbF_2 post-treatment.

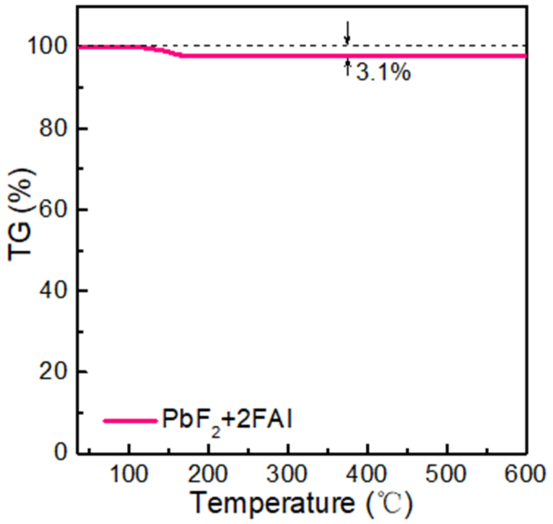

Supplementary Figure 15. TGA heating curves of FAI+PbF_2 powders expressed as weight % as a function of applied temperature.

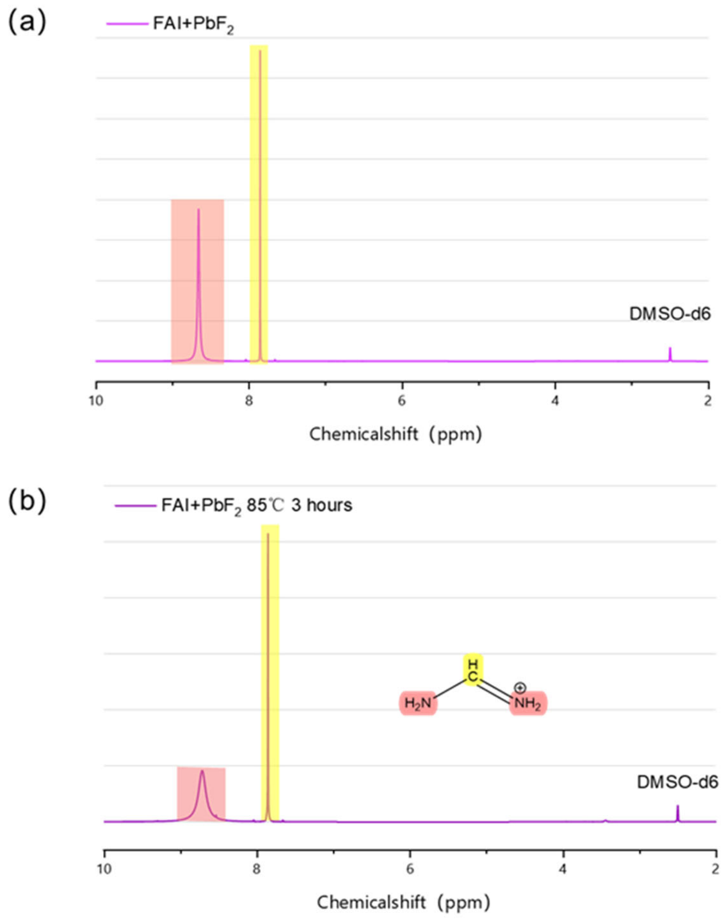

Supplementary Figure 16. The full ^{1}H NMR spectra of (a) FAI+PbF_2 and (b) FAI+PbF_2 aging at 85°C for 3 hours.

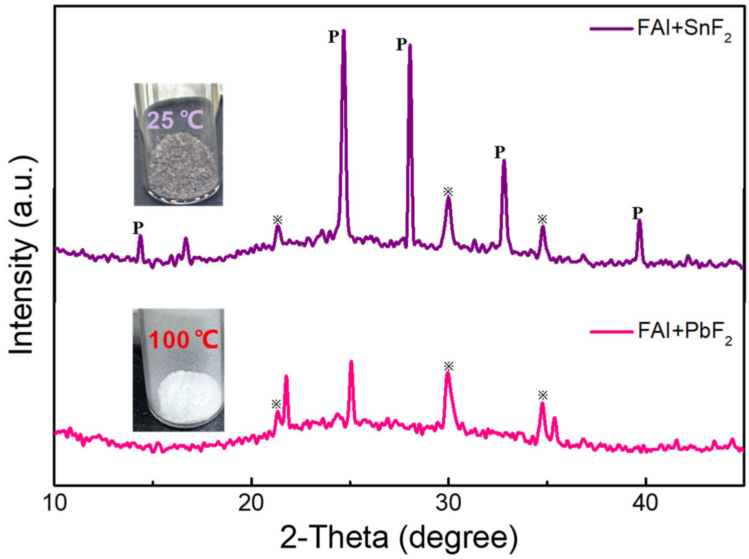

Supplementary Figure 17. XRD patterns of FAI+SnF_2 and FAI+PbF_2 powders heating at 100 °C. Insets: FAI+SnF_2 powder at room temperature which turns to black quickly after shaking; FAI+PbF_2 powder shows no color change during heating from room temperature to 100 °C. P and * represent the peaks of perovskite phase and ITO, respectively.

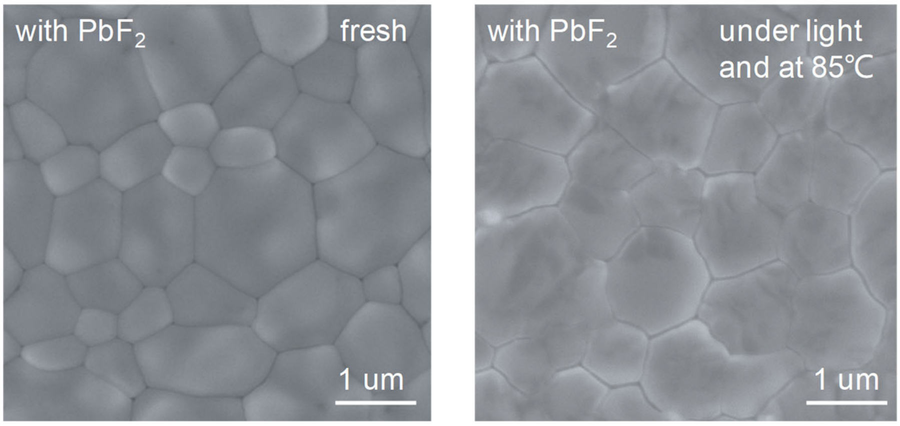

Supplementary Figure 18. SEM spectra of Sn-Pb perovskite film without SnF_2 additive and with PbF_2 post-treatment before and after aging at 85 °C with light soaking for 200 h.

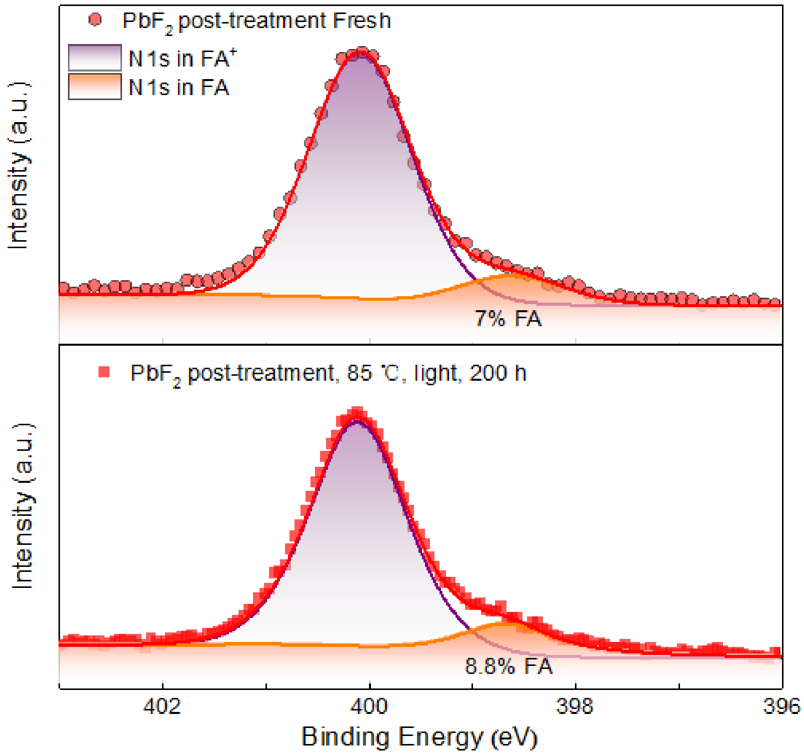

Supplementary Figure 19. N *1s* XPS spectra of Sn-Pb perovskite film without SnF_2 additive and with PbF_2 post-treatment aging at 85 °C with light soaking for 200 h.

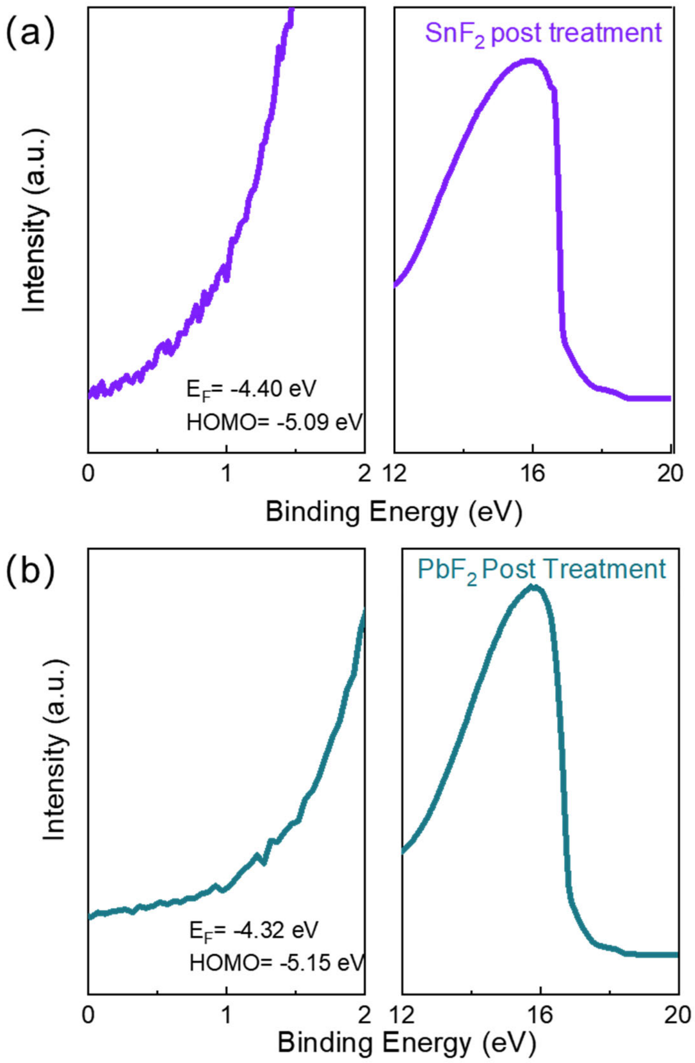

Supplementary Figure 20. The UPS spectra of the perovskite with (a) SnF_2 post-treatment and (b) PbF_2 post-treatment.

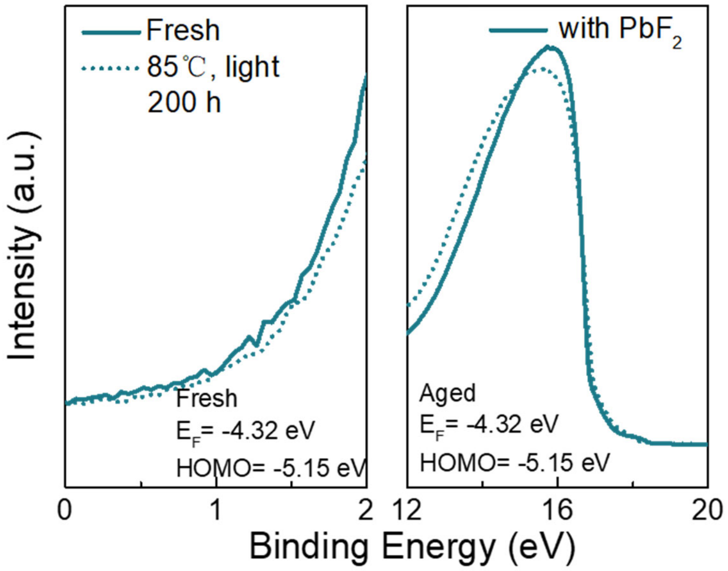

Supplementary Figure 21. UPS of Sn-Pb perovskite without SnF_2 additive and with PbF_2 post-treatment before and after aging at 85 °C with light soaking for 200 h.

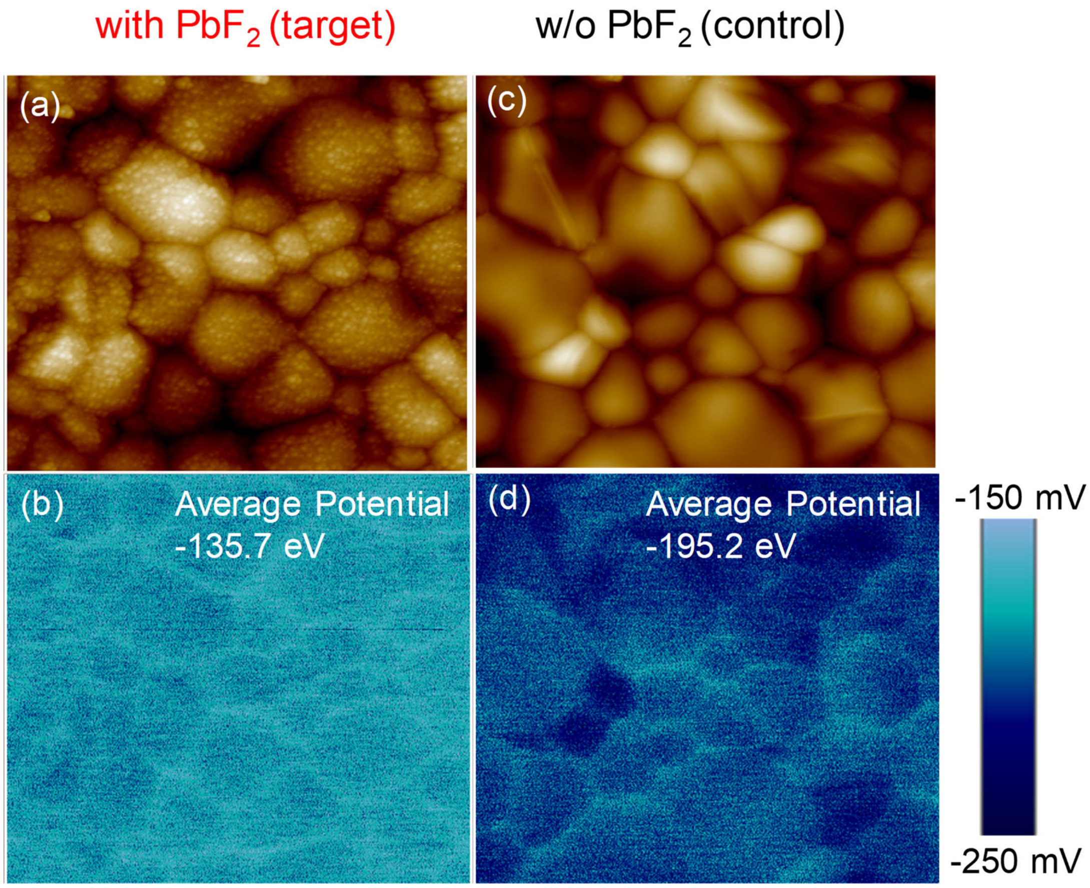

Supplementary Figure 22. The Atomic Force Microscopy (AFM) and Kelvin Probe Force Microscopy (KPFM) of Sn-Pb perovskite film with (a-b) and without (c-d) PbF_2 post-treatment.

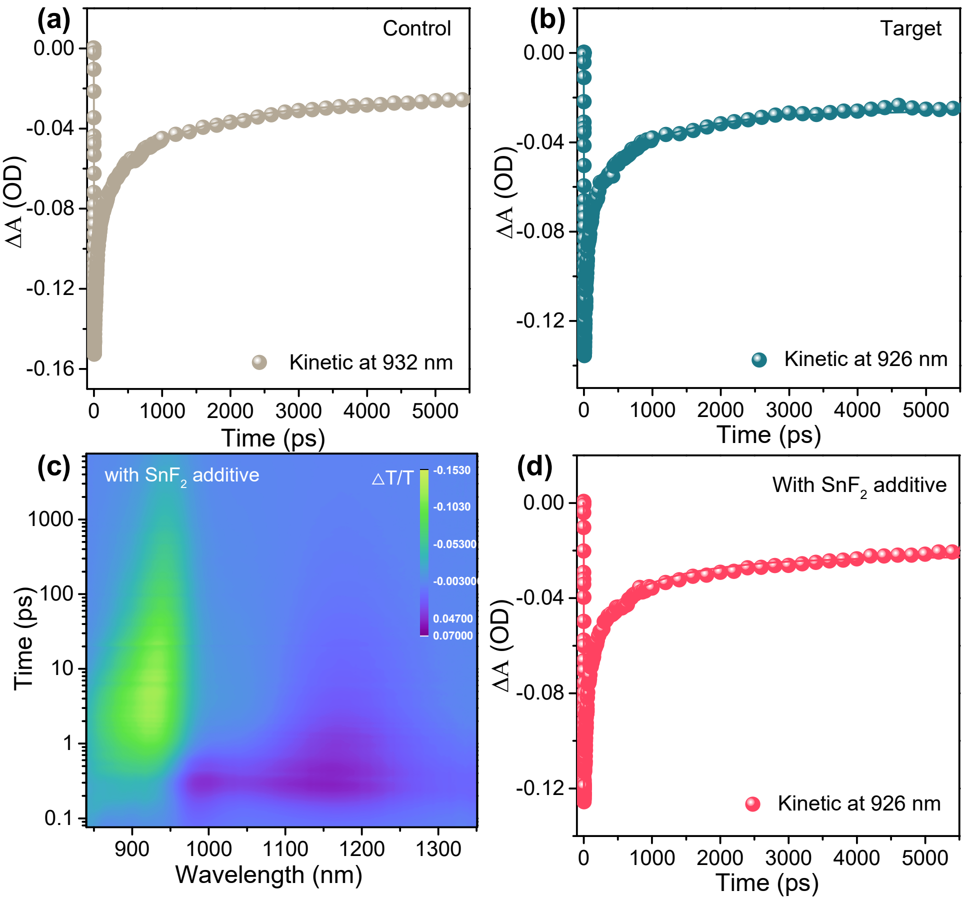

Supplementary Figure 23. The TA pseudo-color spectrum of SnF_2 additive sample and the TA signal decay situations. (a-b) TA signal decay of control (a) and target (b) perovskite films. (c-d) TA spectra and TA signal decay of the perovskite film with SnF_2 additive.

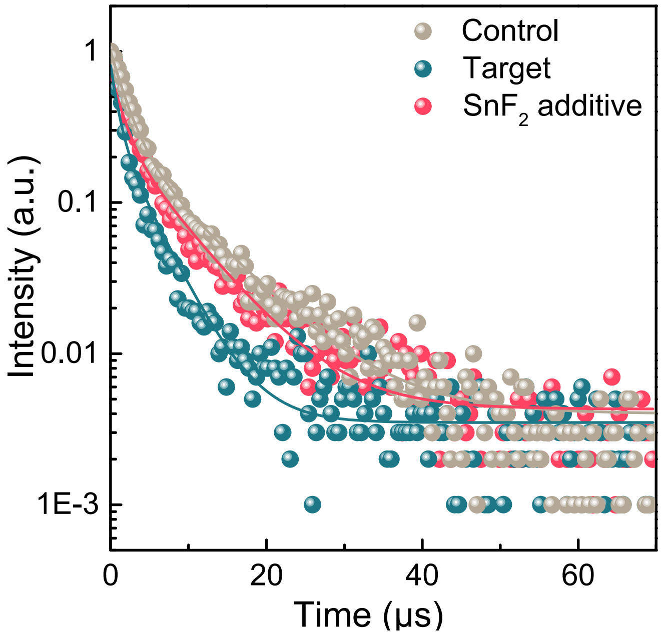

Supplementary Figure 24. TRPL of the control, target and perovskite film with SnF_2 additive. Sample structure: glass/perovskite/C60.

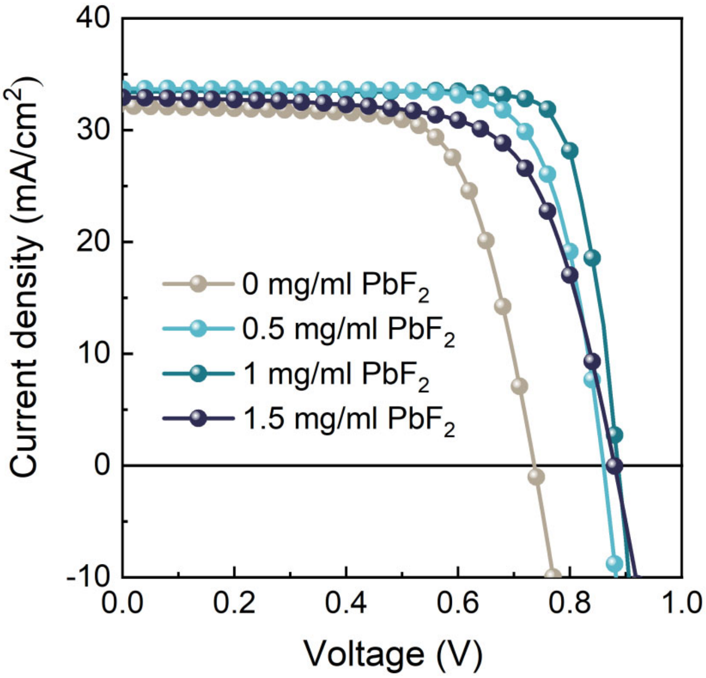

Supplementary Figure 25. The *J-V* curves of SnF_2-free devices post-treated with different concentrations of PbF_2.

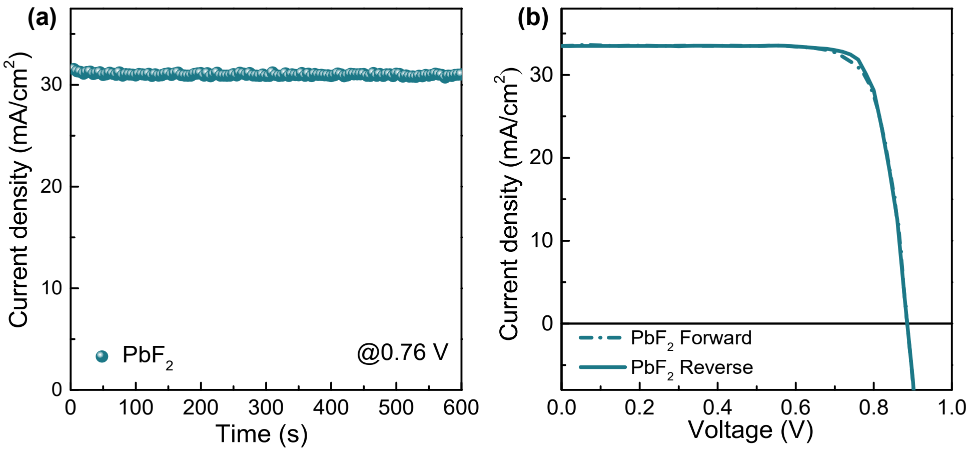

Supplementary Figure 26. The short time MPP and hysteresis characteristics of the target PSC. (a) The steady-state photocurrent output tracking of target device under MPP. (b) The *J*-*V* curves of target devices measured by forward and reverse scans.

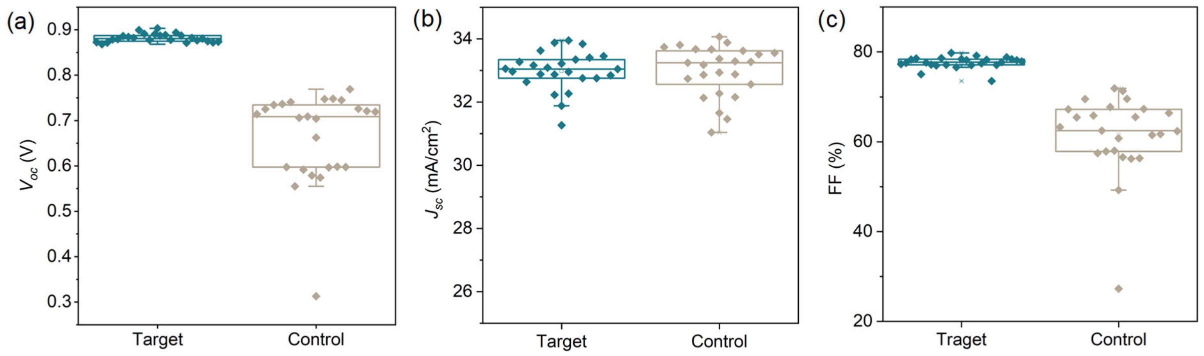

Supplementary Figure 27. The statistical (a) V_{OC}, (b) J_{SC} and (c) FF of 25 randomly selected target and control devices.

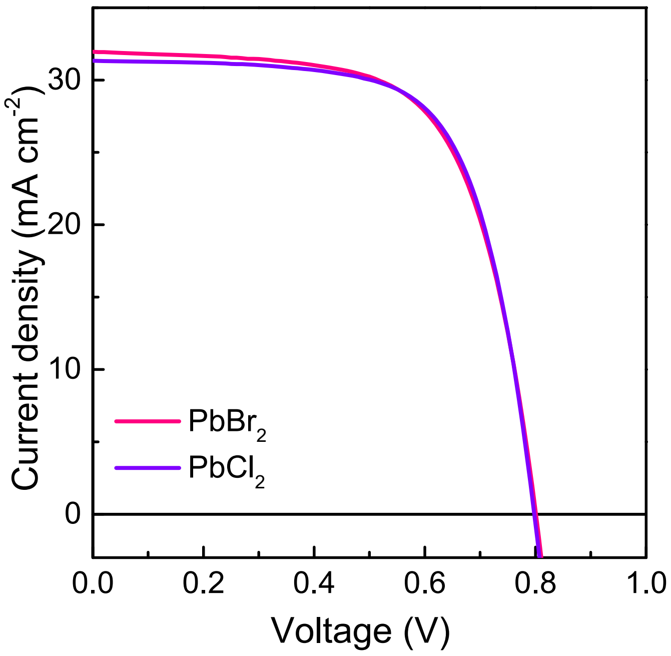

Supplementary Figure 28. The *J-V* curves of SnF_2-free devices with different post-treatments.

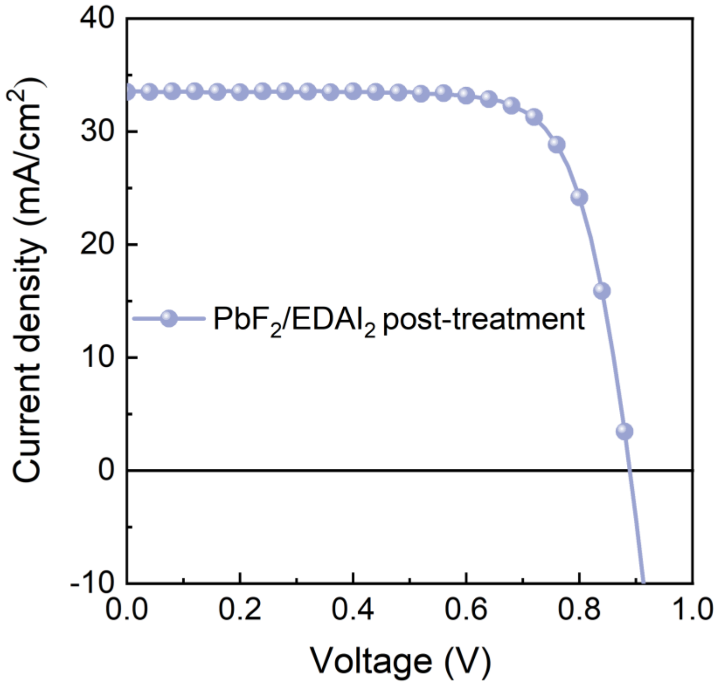

Supplementary Figure 29. The *J-V* curves of Sn-Pb PSCs with post-treatments in combination of PbF_2 and $EDAI_2$.

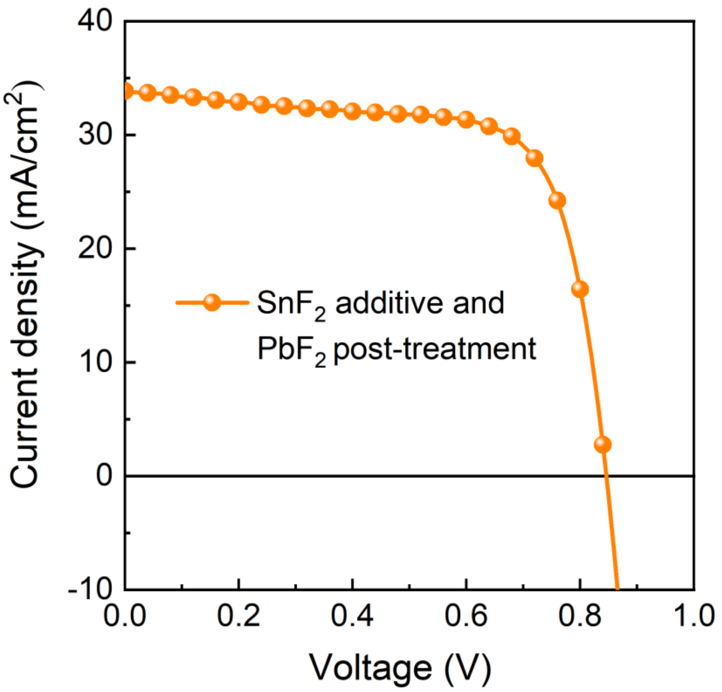

Supplementary Figure 30. The *J*-*V* curves of Sn-Pb PSCs with both SnF_2 additive and PbF_2 post-treatments.

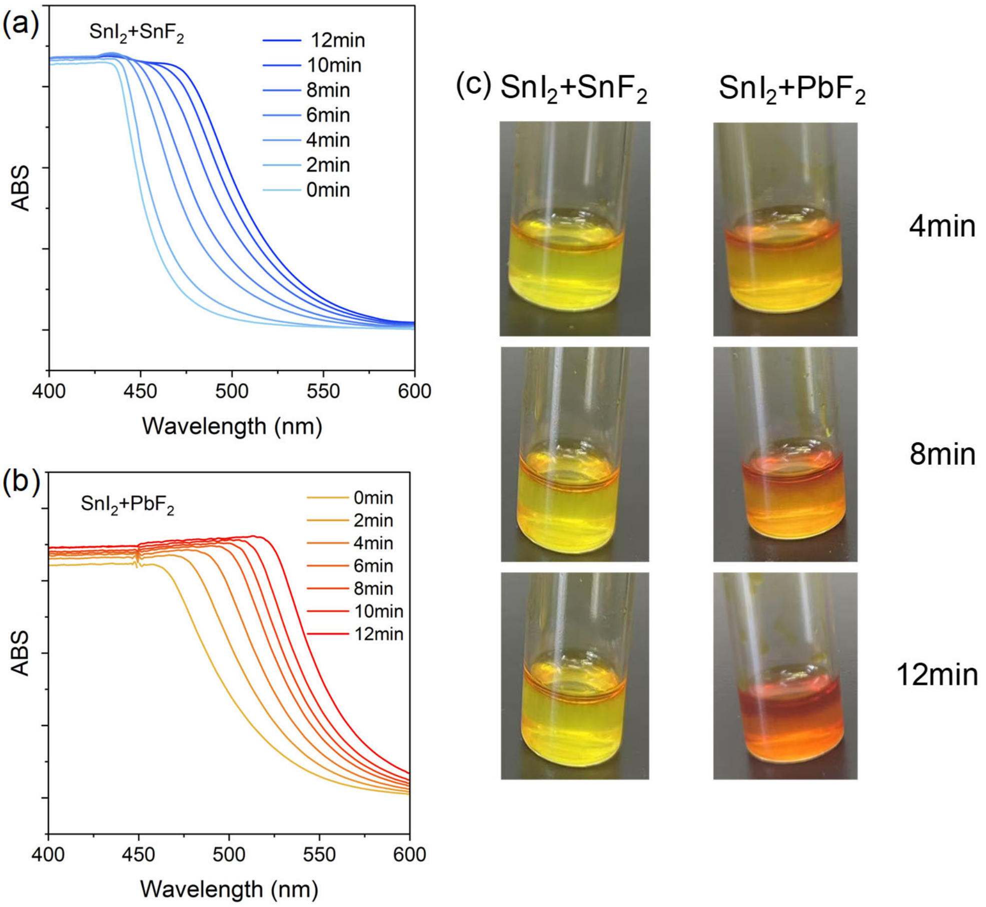

Supplementary Figure 31. The UV-vis absorption spectra of tin-lead perovskite precursor solution with SnF_2 (a) and PbF_2 (b) additive aging in air. (c) The color changes of SnI_2 with SnF_2 and PbF_2 additive.

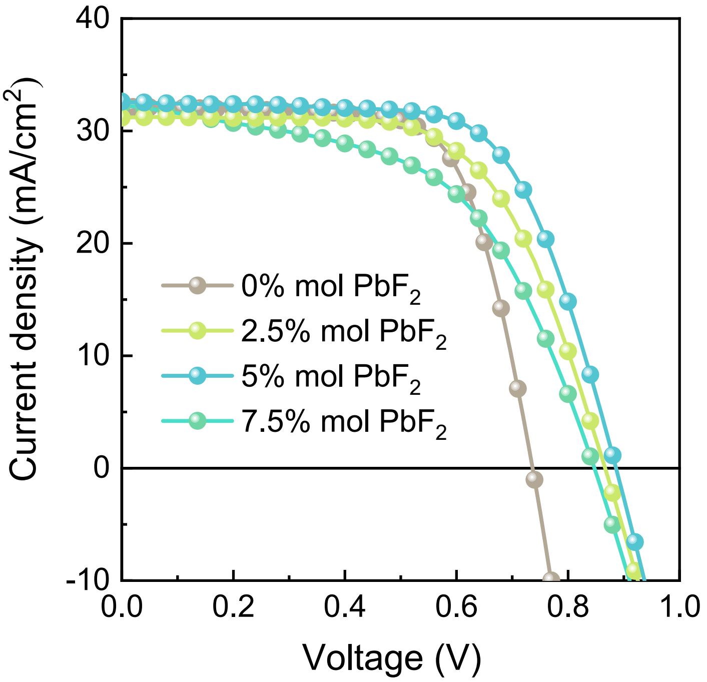

Supplementary Figure 32. *J-V* curves of Sn-Pb PSCs with different concentration PbF_2 additive.

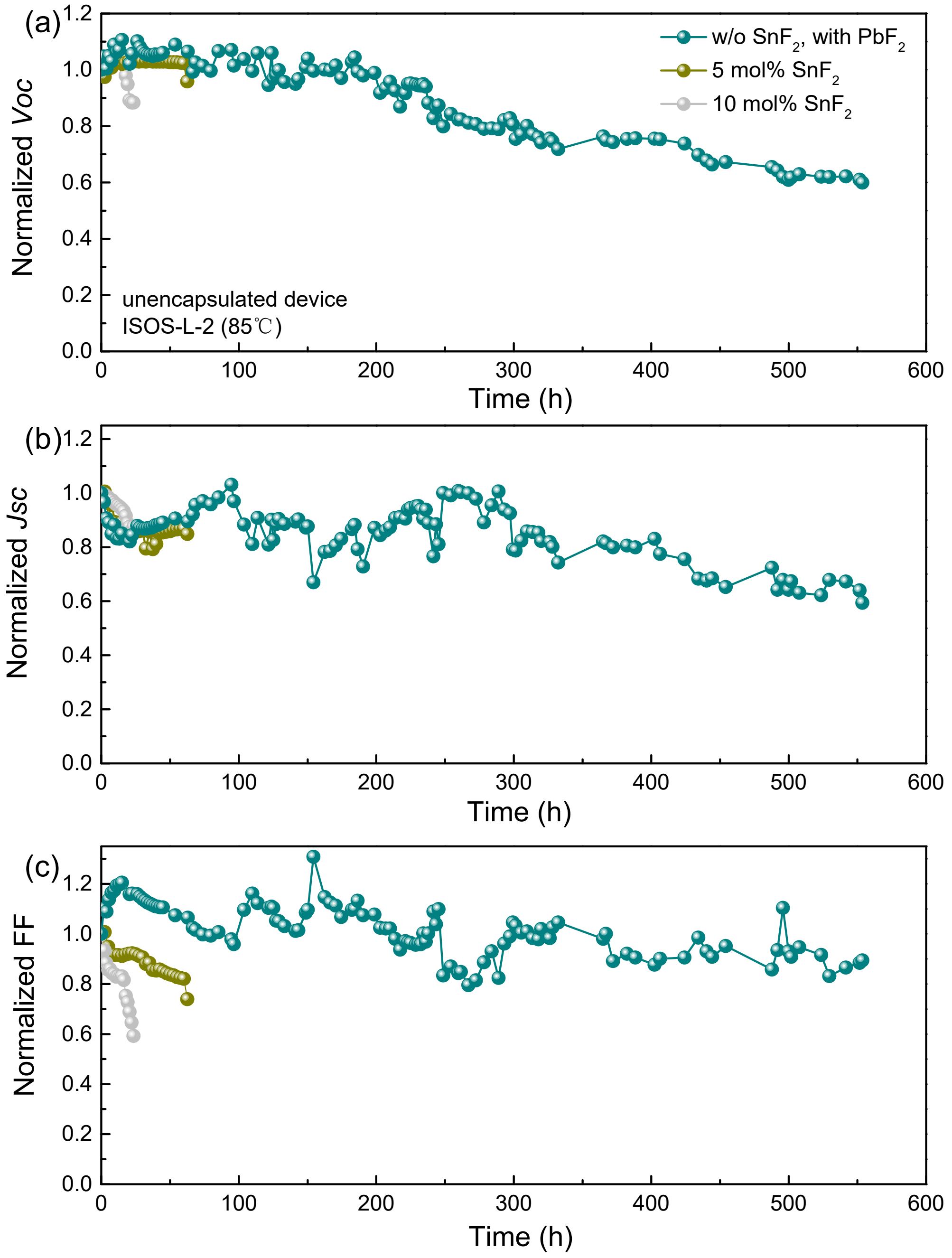

Supplementary Figure 33. The evolution of (a) V_{OC}, (b) J_{SC}, and (c) FF during MPP tracing of unencapsulated devices in N_2 at 85 °C.

Supplementary Table 1. The Pb and Sn atomic concentration and Sn/Pb atomic ration obtained in SEM-EDS mapping.

Sample	Pb (At %)	Sn (At %)	Sn/Pb
Pristine	5.7	6.7	1.17
PbF_2 Post-treatment	5.5	4.4	0.78

Supplementary Table 2. Fitted parameters of the ground-state bleaching kinetics of control, target and SnF_2 doped Sn-Pb perovskite films.

Samples	A_1	τ_1 (ps)	A_2	τ_2 (ps)	A_3	τ_3 (ps)
Control	-0.268	21.1	-0.299	160	-0.268	1330
Target	-0.323	18.5	-0.297	147	-0.206	1130
with SnF_2	-0.380	20.2	-0.318	202	-0.185	1230

Supplementary Table 3. Fitted decay parameters of TRPL of control, target and SnF_2 doped Sn-Pb perovskite films.

Samples	A_1	τ_1 (μs)	A_2	τ_2 (μs)	$T_{average}$ (μs)
Control	0.58	2.436	0.42	11.78	9.70
Target	0.65	1.245	0.35	6.675	5.28
with SnF_2	0.60	2.077	0.40	10.67	8.73

Supplementary Table 4. Summary of photovoltaic performance of SnF_2-free devices post-treated with different concentrations of PbF_2.

PbF_2 conc. (mg/ml)	V_{OC} (V)	J_{SC} (mA/cm^2)	FF (%)	PCE (%)
0	0.730	32.1	69.5	16.40
0.5	0.860	33.6	74.8	21.66
1.0	0.884	33.5	81.3	24.07
1.5	0.879	32.9	67.6	19.59

Supplementary Table 5. Summary of photovoltaic performance of target Sn-Pb perovskite PSCs under forward and reverse scans.

Samples	V_{OC} (V)	J_{SC} (mA·cm^{-2})	FF (%)	PCE (%)
Reverse	0.884	33.49	81.30	24.07
Forward	0.884	33.50	79.49	23.54

Supplementary Table 6. Summary of photovoltaic performance of SnF_2-free Sn-Pb perovskite PSCs with different post-treatments.

Samples	V_{OC} (V)	J_{SC} (mA·cm^{-2})	FF (%)	PCE (%)
$PbCl_2$	0.80	31.34	67.60	16.91
$PbBr_2$	0.80	31.96	65.34	16.73

Supplementary Table 7. Photovoltaic performance of Sn-Pb perovskite PSCs with post-treatment in combination of PbI_2 and $EDAI_2$.

post-treatment	V_{OC} (V)	J_{SC} (mA/cm^2)	FF (%)	PCE (%)
PbF_2/$EDAI_2$	0.888	33.48	75.6	22.5

Supplementary Table 8. Photovoltaic performance of Sn-Pb perovskite with both SnF_2 additive and PbF_2 post-treatments.

	V_{OC} (V)	J_{SC} (mA/cm^2)	FF (%)	PCE (%)
SnF_2 additive and PbF_2 treatment	0.846	33.8	71.0	20.35

Supplementary Table 9. Summary of photovoltaic performance of Sn-Pb perovskite PSCs with different concentration PbF_2 additive.

PbF_2 conc. (mol% vs. perovskite)	V_{OC} (V)	J_{SC} (mA/cm^2)	FF (%)	PCE (%)
0	0.730	32.1	69.5	16.4
2.5	0.866	31.1	62.9	17.02
5.0	0.885	32.6	66.1	19.1
7.5	0.885	34.6	51.7	15.08

Supplementary Table 10. The reported MPP tracing stability of Sn-Pb PSCs at different temperatures. T_n represents the time that device degrades to n% of initial PCE.

Device Structure	MPP Stability	
FTO/PEDOT:PSS/perovskite/C60/BCP/Cu	T_{90}=795 h room temperature	1
ITO/perovskite/C60/SnO_2/IZO	T_{100}=100 h room temperature	2
ITO/PEDOT:PSS/perovskite/C60/BCP/Ag	T_{80}=780 h 25 °C	3
ITO/PEDOT:PSS/perovskite/C60/BCP/Ag	T_{80}=750 h 45 °C	4
ITO/PEDOT:PSS/perovskite/C60/BCP/Cu	T_{90}=128 h 55 °C	5
ITO/PEDOT:PSS/perovskite/C60/BCP/Ag	T_{82}=1830 h 30-35 °C	6
FTO/PEDOT:PSS/perovskite./C60/BCP/Ag	T_{82}=450 h room temperature	7
ITO/perovskite/PCBM/C60/Ag	T_{90}=1000 h room temperature	8
FTO/2PACz/MPA/perovskite/PCBM/C60/BCP/Ag	T_{100}=100 h room temperature	
ITO/PEDOT:PSS/Perovskite/C60/SnO_2/Cu	$T_{91.3}$=800 h room temperature encapsulated	9
ITO/P3CT-Pb/perovskite/C60/BCP/Ag	T_{90}=1000 h room temperature encapsulated	10
ITO/PEDOT:PSS/perovskite/C60/BCP/Ag	T_{92}=450 h room temperature	11
ITO/PEDOT:PSS/perovskite/PCBM/BCP/Ag	T_{98}=150 h 35 °C	12
FTO/PEDOT:PSS/perovskite/C60/BCP/Ag	T_{80}=200 h room temperature	13
ITO/PEDOT:PSS/perovskite/C60/ALD SnO_2/Ag	T_{85}=1000 h 35 °C	14
ITO/PEDOT:PSS/perovskite/C60/SnO_2/Ag	T_{90}=1000 h 25±5 °C	15
ITO/NiO_x/perovskite/PCBM/BCP/Ag	T_{90}=150 h room temperature	16
ITO/PTAA/perovskite/C60/BCP/Ag	T_{90}=1600 h, T_{80}=2700 h room temperature	17
ITO/SnOCl/perovs/PCBM/C60/ALD SnO_2/Cu	T_{87}=1200 h 30 °C<br>T_{81}=850 h 50 °C	18
ITO/neutral PEDOT/SnOCl/perovskite/C60/SnO_2/Cu	T_{85}=1500 h 50 °C	18
ITO/PEDOT:PSS/perovskite/C60/ALD-SnO_2/Ag	T_{90}=650 h 45 °C	19
ITO/P3CT-Cs/perovskite/C60/BCP/LiF/Cu	T_{60}=550 h 85 °C	This work

Supplementary References

1. Zhang, Y., Li, C., Zhao, H., Yu, Z., Tang, X., Zhang, J., Chen, Z., Zeng, J., Zhang, P., Han, L., Chen, H. Synchronized Crystallization in Tin-Lead Perovskite Solar Cells. *Nat. Commun.* **15**, 6887 (2024).
2. Prasanna, R., Leijtens, T., Dunfield, S. P., Raiford, J. A., Wolf, E. J., Swifter, S. A., Werner, J., Eperon, G. E., de Paula, C., Palmstrom, A. F., Boyd, C. C., van Hest, M. F. A. M., Bent, S. F., Teeter, G., Berry, J. J., McGehee, M. D. Design of

Low Bandgap Tin-Lead Halide Perovskite Solar Cells to Achieve Thermal, Atmospheric and Operational Stability. *Nat. Energy* **4**, 939-947 (2019).

3. Lin, Z., Chen, J., Duan, C., Fan, K., Li, J., Zou, S., Zou, F., Yuan, L., Zhang, Z., Zhang, K., Lam, M. Y., Sergeev, A. A., Qiu, J., Wong, K. S., Yan, H., Yan, K. Self-Assembly Homojunction of Sn-Pb Perovskite by Antioxidant for All-Perovskite Tandem Solar Cells with Improved Efficiency and Stability. *Energy Environ. Sci.* **17**, 6314-6322 (2024).
4. Tong, J., Gong, J., Hu, M., Yadavalli, S. K., Dai, Z., Zhang, F., Xiao, C., Hao, J., Yang, M., Anderson, M. A., Ratcliff, E. L., Berry, J. J., Padture, N. P., Zhou, Y., Zhu, K. High-Performance Methylammonium-Free Ideal-Band-Gap Perovskite Solar Cells. *Matter* **4**, 1-12 (2021).
5. Zhou, J., Fu, S., Zhou, S., Huang, L., Wang, C., Guan, H., Pu, D., Cui, H., Wang, C., Wang, T., Meng, W., Fang, G., Ke, W. Mixed Tin-Lead Perovskites with Balanced Crystallization and Oxidation Barrier for All-Perovskite Tandem Solar Cells. *Nat. Commun.* **15**, 2324 (2024).
6. Kumar, M. H., Dharani, S., Leong, W. L., Boix, P. P., Prabhakar, R. R., Baikie, T., Shi, C., Ding, H., Ramesh, R., Asta, M., Graetzel, M., Mhaisalkar, S. G., Mathews, N. Lead-Free Halide Perovskite Solar Cells with High Photocurrents Realized through Vacancy Modulation. *Adv. Mater.* **26**, 7122-7127 (2014).
7. Savill, K. J., Ulatowski, A. M., Farrar, M. D., Johnston, M. B., Snaith, H. J., Herz, L. M. Impact of Tin Fluoride Additive on the Properties of Mixed Tin-Lead Iodide Perovskite Semiconductors. *Adv. Funct. Mater.* **30**, 2005594 (2020).
8. Yuan, F., Folpini, G., Liu, T., Singh, U., Treglia, A., Lim, J. W. M., Klarbring, J., Simak, S. I., Abrikosov, I. A., Sum, T. C., Petrozza, A., Gao, F. Bright and Stable Near-Infrared Lead-Free Perovskite Light-Emitting Diodes. *Nat. Photon.* **18**, 170-176 (2024).
9. Chen, Q., Luo, J., He, R., Lai, H., Ren, S., Jiang, Y., Wan, Z., Wang, W., Hao, X., Wang, Y., Zhang, J., Constantinou, I., Wang, C., Wu, L., Fu, F., Zhao, D. Unveiling Roles of Tin Fluoride Additives in High-Efficiency Low-Bandgap Mixed Tin-Lead Perovskite Solar Cells. *Adv. Energy Mater.* **11**, 2101045 (2021).

10. Treglia, A., Ambrosio, F., Martani, S., Folpini, G., Barker, A.J., Albaqami, M. D., Angelis, F. D., Poli, I., Petrozza, A. Effect of Electronic Doping and Traps on Carrier Dynamics in Tin Halide Perovskites. *Mater. Horiz.* **9**, 1763-1773 (2022).
11. Tong, J., Jiang, Q., Ferguson, A. J., Palmstrom, A. F., Wang, X., Hao, J., Dunfield, S. P., Louks, A. E., Harvey, S. P., Li, C., Lu, H., France, R. M., Johnson, S. A., Zhang, F., Yang, M., Geisz, J. F., McGehee, M. D., Beard, M. C., Yan, Y., Kuciauskas, D., Berry, J. J., Zhu, K. Carrier control in Sn-Pb Perovskites via 2D Cation Engineering for All-Perovskite Tandem Solar Cells with Improved Efficiency and Stability. *Nat. Energy* **7**, 642-651 (2022).
12. Kapil, G., Bessho, T., Sanehira, Y., Sahamir, S. R., Chen, M., Baranwal, A. K., Liu, D., Sono, Y., Hirotani, D., Nomura, D., Nishimura, K., Kamarudin, M. A., Shen, Q., Segawa, H., Hayase, S. Tin-Lead Perovskite Solar Cells Fabricated on Hole Selective Monolayers. *ACS Energy Lett.* **7**, 966-974 (2022).
13. Yan, W., Li, C., Peng, C., Tan, S., Zhang, J., Jiang, H., Xin, F., Yue, F., Zhou, Z. Hot-carrier Cooling Regulation for Mixed Sn-Pb Perovskite Solar Cells. *Adv. Mater.* **36**, 2312170 (2024).
14. Hu, S., Otsuka, K., Murdey, R., Nakamura, T., Truong, M. A., Yamada, T., Handa, T., Matsuda, K., Nakano, K., Sato, A., Marumoto, K., Tajima, K., Kanemitsu, Y., Wakamiya, A. Optimized Carrier Extraction at Interfaces for 23.6% Efficient Tin-Lead Perovskite Solar Cells. *Energy Environ. Sci.* **15**, 2096-2107 (2022).
15. Tan, S., Li, C., Peng, C., Yan, W., Bu, H., Jiang, H., Yue, F., Zhang, L., Gao, H., Zhou, Z. Sustainable Thermal Regulation Improves Stability and Efficiency in All-Perovskite Tandem Solar Cells. *Nat. Commun.* **15***,* 4136 (2024).
16. Cao, J., Loi, H.-L., Xu, Y., Guo, X., Wang, N., Liu, C.-k., Wang, T., Cheng, H., Zhu, Y., Li, M. G., Wong, W.-Y., Yan, F. High-Performance Tin-Lead Mixed-Perovskite Solar Cells with Vertical Compositional Gradient. *Adv. Mater.* **34**, 2107729 (2022).
17. Liang, Z., Xu, H., Zhang, Y., Liu, G., Chu, S., Tao, Y., Xu, X., Xu, S., Zhang, L., Chen, X., Xu, B., Xiao, Z., Pan, X., Ye, J. Selective Targeting Anchor Strategy Afford Efficient and Stable Ideal Bandgap Perovskite Solar Cells. *Adv. Mater.* **34**, 2110241 (2022).

18. Yu, Z., Wang, J., Chen, B., Uddin, M. A., Ni, Z., Yang, G., Huang, J. Solution Processed Ternary Tin (II) Alloy as Hole-Transport Layer of Sn-Pb Perovskite Solar cells for Enhanced Efficiency and Stability. *Adv. Mater.* **34**, 2205769 (2022).
19. Yu, D., Pan, M., Liu, G., Jiang, X., Wen, X., Li, W., Chen, S., Zhou, W., Wang, H., Lu, Y., Ma, M., Zang, Z., Cheng, P., Ji, Q., Zheng, F., Ning, Z. Electron-Withdrawing Organic Ligand for High-Efficiency All-Perovskite Tandem Solar Cells. *Nat. Energy* **9**, 298-307 (2024).
