# Supplementary material for: A tin fluoride-free, efficient and durable tin-lead perovskite solar cell
Source: Nat Commun. 2026 Jan 12;17:360. doi: 10.1038/s41467-025-65445-0 (PMC12796181; doi:10.1038/s41467-025-65445-0)
Supplement: Supplementary file 2 — Reporting Summary [file 41467_2025_65445_MOESM2_ESM.pdf]

nature portfolio

Corresponding author(s): Wenxiao Zhang, Junfeng Fang, Feng Gao

Solar Cells Reporting Summary

Nature Portfolio wishes to improve the reproducibility of the work that we publish. This form is intended for publication with all accepted papers reporting the characterization of photovoltaic devices and provides structure for consistency and transparency in reporting. Some list items might not apply to an individual manuscript, but all fields must be completed for clarity.

For further information on Nature Research policies, including our data availability policy, see Authors & Referees.

Experimental design

Please check the following details are reported in the manuscript, and provide a brief description or explanation where applicable.

1. Dimensions

Area of the tested solar cells
- [x] Yes — The device area defined by the mask was 0.0836 cm2.
- [ ] No — *Explain why this information is not reported/not relevant.*

Method used to determine the device area
- [x] Yes — The active areas of the solar cells were defined by the overlapping area of ITO and silver electrodes.
- [ ] No — *Explain why this information is not reported/not relevant.*

2. Current-voltage characterization

Current density-voltage (J-V) plots in both forward and backward direction
- [x] Yes — Figure 5a and Supplementary Figure 26.
- [ ] No

Voltage scan conditions
- [x] Yes — The J-V curves were measured from 1.00 V to -0.2 V with a scanning rate of 100 mV/s (voltage step of 20 mV).
- [ ] No — *Explain why this information is not reported/not relevant.*

Test environment
- [x] Yes — The devices were measured in the glovebox with N2 filling at room temperature.
- [ ] No — *Explain why this information is not reported/not relevant.*

Protocol for preconditioning of the device before its characterization
- [ ] Yes — *Provide a description of the protocol.*
- [x] No — No preconditioning was used for the devices.

Stability of the J-V characteristic
- [x] Yes — Maximum power point was verified as shown in Supplemental Figure 26a.
- [ ] No — *Explain why this information is not reported/not relevant.*

3. Hysteresis or any other unusual behaviour

Description of the unusual behaviour observed during the characterization
- [ ] Yes — *Provide a description of hysteresis or any other unusual behaviour observed during the characterization.*
- [x] No — We observed negligible hystersis in the devices as shown in Supplementary Figure 26.

Related experimental data
- [x] Yes — Supplementary Figure 26
- [ ] No — *Explain why this information is not reported/not relevant.*

4. Efficiency

External quantum efficiency (EQE) or incident photons to current efficiency (IPCE)
- [x] Yes — Figure 5b.
- [ ] No — *Explain why this information is not reported/not relevant.*

A comparison between the integrated response under the standard reference spectrum and the response measure under the simulator
- [x] Yes — The integrated Jsc values from EQE were consistent with Jsc values from J-V measurements (Figure 5a and 5b).
- [ ] No — *Explain why this information is not reported/not relevant.*

For tandem solar cells, the bias illumination and bias voltage used for each subcell

☐ Yes
☒ No

Provide a description of the measurement conditions.

We have not report tandem solar cell device performance in this work.

5. Calibration

Light source and reference cell or sensor used for the characterization

☒ Yes
☐ No

Characteristics of films and devices: The standard solar cells were used to check the spectra of solar simulator and EQE equipments.

Explain why this information is not reported/not relevant.

Confirmation that the reference cell was calibrated and certified

☒ Yes
☐ No

The standard solar cell was used as the reference for calibration.

Explain why this information is not reported/not relevant.

Calculation of spectral mismatch between the reference cell and the devices under test

☒ Yes
☐ No

The spectral mismatch is negligible as the difference between the Jsc and EQE results.

Explain why this information is not reported/not relevant.

6. Mask/aperture

Size of the mask/aperture used during testing

☒ Yes
☐ No

The area of mask was 0.0836 cm2.

Explain why this information is not reported/not relevant.

Variation of the measured short-circuit current density with the mask/aperture area

☒ Yes
☐ No

EQE curves in Figure 5b.

Explain why this information is not reported/not relevant.

7. Performance certification

Identity of the independent certification laboratory that confirmed the photovoltaic performance

☐ Yes
☒ No

Identify the independent certification laboratory.

We did not get an independent certification.

A copy of any certificate(s)

☐ Yes
☐ No

Certificate copies should be provided in the Supplementary information. Please state the supplementary item number.

Explain why this information is not reported/not relevant.

8. Statistics

Number of solar cells tested

☒ Yes
☐ No

Figure 5c and Supplementary Figure 27 shows the PCE of 25 individual control and targeted devices.

Explain why this information is not reported/not relevant.

Statistical analysis of the device performance

☒ Yes
☐ No

Figure 4c.and Supplementary Figure 27.

Explain why this information is not reported/not relevant.

9. Long-term stability analysis

Type of analysis, bias conditions and environmental conditions

☒ Yes
☐ No

The MPP stability test was conducted on encapsulated devices in N2 under 1-sun equivalent illumination (white light-emitting diodes) at 85 ? which was monitored by an infrared thermometer.

Explain why this information is not reported/not relevant.
